# The Efficacy of Artecoll Injections for the Augmentation of Nipple Projection in Breast Reconstruction

**Published:** 2010-01-04

**Authors:** Colleen M. McCarthy, Nancy VanLaeken, Peter Lennox, Amie M. Scott, Andrea L. Pusic

**Affiliations:** ^a^ Plastic and Reconstructive Surgery Service, Department of Surgery, Memorial Sloan-Kettering Cancer Center, New York; ^b^ Division of Plastic and Reconstructive Surgery, Department of Surgery, University of British Columbia, Vancouver, British Columbia, Canada

## Abstract

**Introduction:** Various techniques have been used in an attempt to achieve long-term nipple projection following nipple-areolar reconstruction. A common setback, however, is the diminution of projection overtime; this phenomenon is particularly evident following implant-based breast reconstruction. Artecoll may be suitable for injection into the nipple complex to maintain permanent, 3-dimensional projection. Artecoll is an injectable substance that is biocompatible and immunologically inert and resists degradation in vivo. The purpose of this study was thus to prospectively evaluate the efficacy of Artecoll (polymethylmethacrylate microspheres suspended in 3.5% denatured bovine collagen with 0.3% lidocaine) in obtaining and maintaining nipple projection following postmastectomy, nipple-areolar reconstruction. **Methods:** A prospective, clinical trial was performed. Consecutive patients deemed to have inadequate nipple projection at least 6 months following “C-V flap” or “modified-skate flap” reconstruction were identified. Only women who had postmastectomy reconstruction with tissue expanders and implants were considered eligible for participation. Artecoll was injected under the nipple at 2 time points: baseline and 3 months. Calipers were used to measure nipple projection preinjection and postinjection at baseline, 3, 6, and 9 months. **Results:** Thirty-three nipples were injected in 23 patients. There were no adverse events. Prior to injection, mean nipple projection was 1.33 ± 1.0 mm. The mean increase in projection over the 9-month study period was both clinically and statistically significant (1.60 ± 1.24 mm; *P* <.001). A history of prior irradiation was a significant negative predictor of final nipple projection (*P* = .012). **Conclusion:** Artecoll injection is both feasible and effective in increasing and maintaining nipple projection in the setting of implant-based breast reconstruction.

By transforming the surgically created breast mound into a more natural-appearing breast, nipple-areolar reconstruction can significantly enhance patients' perspective of their overall aesthetic result and can improve patient satisfaction.^1^^-^3 The most challenging aspect of nipple reconstruction is, however, the creation of a 3-dimensional structure with the dimensions and contour similar to a natural nipple. Various techniques have been used in an attempt to achieve long-term nipple projection, including local skin flaps, cartilage and fascial grafts, and nipple-sharing techniques. Irrespective of the technique used, a common disappointment is the loss of projection overtime.^4^^-^6 This phenomenon is particularly evident following implant-based breast reconstruction.^7^

As a means to increase projection after nipple reconstruction, the subcutaneous injection of Artecoll may be useful. Artecoll (Artes Medical, San Diego, Calif; Canderm Pharma Inc, Saint-Laurent, Canada) is an injectable substance that consists of inert, nonbiodegradable poly(methyl methacrylate) microspheres suspended in a partially denatured 3.5% bovine collagen.^8^ After the subcutaneous injection of Artecoll, the collagen carrier is degraded by the body within 3 months and completely replaced by a matrix of autogenous collagen at a similar rate. Because the microspheres are nonbiodegradable and too large to migrate to be phagocytosed by macrophages, tissue augmentation is expected to be permanent.^9^^-^11 Thus, it is hypothesized that the subcutaneous injection of Artecoll will provide long-lasting nipple projection with minimal patient morbidity and create a more natural-looking breast following postmastectomy reconstruction.

The purpose of this study is thus to prospectively evaluate the efficacy of Artecoll in augmenting and maintaining nipple projection in the setting of postmastectomy, implant reconstruction.

## METHODS

A prospective, clinical trial was designed. Formal approval by the institutional review board was obtained. Consecutive patients deemed to have inadequate nipple projection at least 6 months following “C-V flap” or “modified-skate flap” reconstruction were identified. Only women who have had postmastectomy reconstruction with tissue expanders and implants were considered eligible for participation. Participants were excluded from participation if they were younger than 21 years and had a known susceptibility to keloid formation and/or a lidocaine, collagen, or dietary beef sensitivity.

Skin testing for collagen sensitivity was performed at least 1 month prior to study initiation. In the event of an adverse reaction, patients were excluded from further participation. In all eligible patients, Artecoll was injected under the nipple at 2 time points: baseline and 3 ± 1 months. Injection with up to 2 syringes (1.4 mL) of Artecoll or injection to “tissue tolerance” was performed at each time point. One clinician performed all of the injections.

Calipers were used to measure nipple projection preinjection and postinjection at baseline and 3 ± 1 months. Follow-up measurements were repeated at 6 ± 1 and 9 ± 1 months. A single investigator was responsible for the performance of all caliper measurements. Photographic documentation was similarly performed.

## Sample size considerations

A priori sample size calculation was performed on the basis of the variance of paired differences obtained from pilot data.^12^ To detect a clinically significant increase in projection (ie, an increase in nipple projection of 200% greater than baseline) with 90% power and a significance level of 0.05, it was determined that 33 nipples were needed.

## Statistical methods

Pairwise differences in projection were calculated using the Wilcoxon signed rank test. Univariate and multivariate linear regression analyses were performed on all variables to determine their influence on the efficacy of Artecoll injection. Statistical significance was set at the 0.05 level. Stata statistical software was used (Stata Corp, College Station, Tex).

## RESULTS

Thirty-three nipples were injected in 23 patients. Mean time from completion of nipple reconstruction was 32.9 ± 20.2 months (range = 7.28–66.07 months). Mean volume injected at each time point was 0.55 ± 0.17 mL (range = 0.2–1.4 mL). There were no adverse events. All patients tolerated the procedure without the need for additional local anesthesia. Transient nipple swelling was noted immediately following injection.

**Figure 1:**
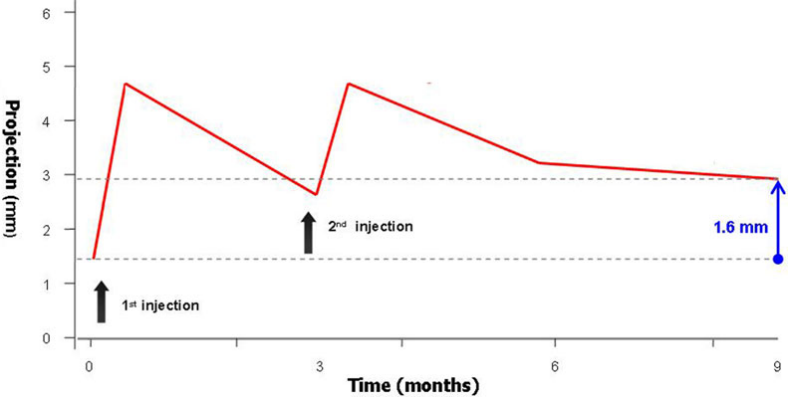
Mean nipple projection.

Prior to injection, mean nipple projection was 1.33 ± 1.0 mm (range = 0.0–4.0 mm). At 3 months postinjection, mean projection was 2.87 ± 1.8 mm (range = 0.0–6.0 mm). At 6 and 9 months, mean projection was 3.09 ± 1.6 mm (range = 0.0–6.0 mm) and 2.93 ± 1.49 mm, respectively (Fig 1).

**Figure 2:**
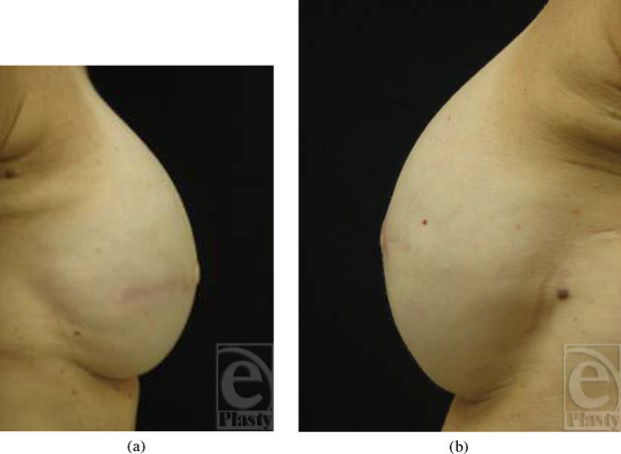
Baseline (a) right breast and (b) left breast.

**Figure 3:**
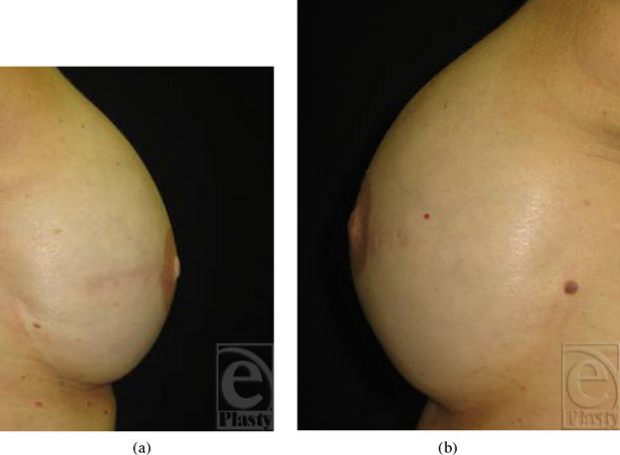
Nine months following Artecoll injections at baseline and 3 months. Nipple areolar tattooing has been completed: (a) right breast and (b) left breast.

**Figure 4:**
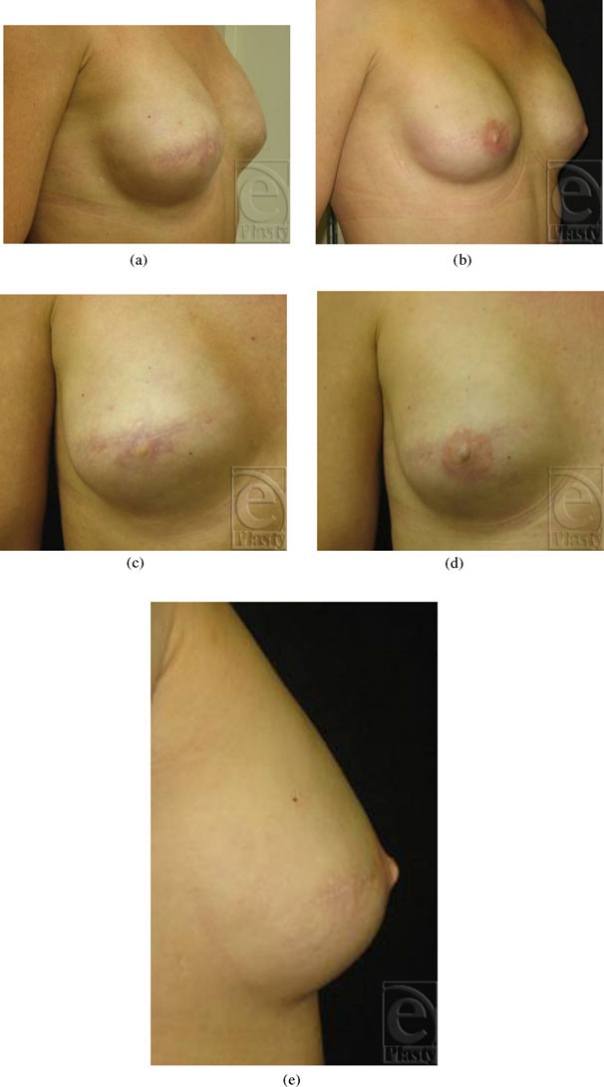
(a) Preoperative oblique view, (b) 9 months following Artecoll injections at baseline and 3 months. Nipple areolar tattooing has been completed, (c) closeup view of baseline nipple projection right breast, (d) closeup anterioposterior view right breast at 9-month follow-up, and (e) lateral view of right nipple.

The mean increase in projection over the 9-month study period was both clinically and statistically significant (1.60 ± 1.24 mm; range = −1.0–4.5 mm) (*P* <.001) (Figs 2–4). The mean increase in projection following the first injection (1.39 ± 1.55 mm; range = −1.0– 4.0 mm; *n* = 31) was significantly higher than that following the second injection (0.43 ± 1.28 mm; range = −2.0–4.0 mm; *n* = 27) (*P* = .0383). Mean final projection (9 months) was significantly lower in the subset of reconstructions that were previously radiated (0.33 ± 0.98 mm; range = 0.0–4.0 mm; *n* = 7) compared with those that had no history of radiation (1.90 ± 1.10 mm; range = 1.0–2.0 mm) (*P* = .005).

The results of multivariate analysis similarly demonstrated that a history of prior chest wall irradiation was a significant negative predictor of total increase in nipple projection (*P* = .012). Conversely, baseline nipple projection, time since completion of nipple reconstruction, and volume of Artecoll injected were not significant predictors of total nipple projection achieved (Tables 1 and 2).

## DISCUSSION

On the basis of the results of this study, it appears that the subcutaneous injection of Artecoll is safe, feasible, and effective in increasing nipple projection after unsatisfactory reconstruction in patients with breast implants. This objective data can now be used to assist patients and surgeons alike in evaluating options when faced with disappointing results after nipple reconstruction with local flaps. In addition, these measures can provide a baseline for comparison following refinements in both surgical and nonoperative techniques.

It has been hypothesized that following nipple reconstruction with local skin flaps, the loss of projection overtime is due to the absence of rigid connective tissue support and/or the imposition of centrifugal, wound contractile forces at the base of the reconstruction.^13^ By injecting Artecoll into the subcutaneous tissues, however, support for the overlying dermal envelope can be restored. Furthermore, while the injected collagen carrier in Artecoll is degraded by the body within a period of 3 months, it is completely replaced by autogenous collagenous tissue, which renders the results from injection long-lasting.

Perhaps not surprisingly, the results of our current study suggest that while the subcutaneous injection of Artecoll can be successfully performed, it appears to be less effective in augmenting the nipple in the setting of prior radiotherapy. It is likely that any radiation-induced skin changes would render the local skin flaps used to reconstruct the nipple less pliable. It follows that the limited laxity of the taut dermal envelope would, in turn, restrict the augmentation achieved through the injection of Artecoll.

These findings are consistent with those reported by Evans et al.^13^ Their group performed a pilot study evaluating the use of a semipermanent soft-tissue filler, calcium hydroxylapatite (Radiesse, Bioform Inc, San Mateo, CA), to maintain and/or restore projection in 6 breast-reconstruction patients. Injections were performed in all 6 patients, 1 year following autogenous tissue or combined autogenous tissue/implant reconstruction. A moderate improvement in nipple appearance postinjection was observed in the majority of cases; yet, in reconstructed nipples with a tighter dermal envelope, the filler injected tended to disperse into the areola creating a convexity to the areola without a discernible increase in nipple projection.

While our current results suggest that the subcutaneous injection of Artecoll results in both a significant and sustained increase in nipple projection in the majority of patients who have undergone prior implant-based reconstruction, these results are not generalizable to those patients who have had autogenous tissue reconstruction. In fact, the results of a pilot study performed by our group would suggest that the subcutaneous injection of Artecoll produces neither a significant nor a sustained increase in nipple projection in this cohort of patients. Future investigation may be warranted.

It is also important to note that the follow-up data in this current study are limited to 9 months following the first injection. Thus, to provide objective evidence of effectiveness of this intervention over the lifetime of the patient, evaluation of the longer-term sustainability of these results must be undertaken. Similarly, the impact of nipple projection on postoperative patient satisfaction must be fully delineated. In breast surgery, the patient's perception of the impact of the reconstruction is the most significant outcome variable and should be rigorously assessed in studies that attempt to evaluate surgical success. Future clinical research should therefore include patient-reported outcome data, such as patient satisfaction with improved nipple projection. Finally, as healthcare costs continue to escalate, there is a growing emphasis on evaluating the cost-effectiveness of an intervention. The high cost of soft-tissue fillers, such as Artecoll, underscores the need to rigorously evaluate these outcomes. The availability of such information could then be used in combination with the objective findings presented here to determine the overall cost utility of Artecoll injections in the setting of unsatisfactory nipple reconstruction.

## CONCLUSION

The subcutaneous injection of Artecoll results in a significant and sustained increase in nipple projection following implant-based breast reconstruction.

## ACKNOWLEDGMENT

Support for this project was provided by the Canadian Society of Plastic Surgeons' Educational Foundation.

## Figures and Tables

**Table 1:** Univariate predictors of change in nipple projection following Artecoll injection

Variable	Coefficient	*P*
Prior history of radiation	−1.558	.005
Time since nipple reconstruction, mo	0.010	.322
Baseline nipple projection	−0.343	.127
Volume per injection, mL	2.839	.031
Total volume injected, mL	0.153	.812

**Table 2:** Multivariate predictors of change in nipple projection following Artecoll injection

Variable	Coefficient	*P*
Prior history of radiation	−1.350	.012
Volume per injection, mL	2.060	.094
